# Supplementary material for: Cloud BioLinux: pre-configured and on-demand bioinformatics computing for the genomics community
Source: BMC Bioinformatics. 2012 Mar 19;13:42. doi: 10.1186/1471-2105-13-42 (PMC3372431; doi:10.1186/1471-2105-13-42)
Supplement: Additional file 1 — Supplementary 1 Cloud BioLinux software documentation in the form of a mini, self-contained website. Users need to download and uncompress the .zip file, and open through a web browser the "index.html" file available on the main directory. (ZIP 1823 kb). [file 1471-2105-13-42-S1.ZIP › Cloud-BioLinux-Package-Documentation/docs/OligoArray.html]

Bio-Linux Software Documentation Pages

Back to search form

## OligoArray

|  |  |
| --- | --- |
| Name | OligoArray |
| Description | **OligoArray** computes gene specific oligonucleotides for genome-scalle oligonucleotide microarray contruction.   Three major criteria are used for selection:  - oligonucleotide melting temperature - specificity to a single target, or at least to the shortest list of possible targets - the inability to fold to form a stable secondary structure at the hybridization temperature.  As of version 2.1, OligoArray depends on the OligoArrayAux sofftware to predict the formation of stable secondary structures.  **References:**  Rouillard JM, Zuker M, Gulari E.OligoArray 2.0: design of oligonucleotide probes for DNA microarrays using a thermodynamic approach. Nucleic Acids Res. 2003 Jun 15;31(12):3057-62.Entrez    Rouillard JM, Herbert CJ, Zuker M. OligoArray: genome-scale oligonucleotide design for microarrays. Bioinformatics. 2002 Mar;18(3):486-7. Entrez. |
| Homepage | http://berry.engin.umich.edu/oligoarray2\_1/ |
| Remote Documentation | http://berry.engin.umich.edu/oligoarray2\_1/ |
